# Trajectories of psychosocial functioning across maltreatment levels: A group-based modeling approach to resilience

**DOI:** 10.1017/S0954579425100758

**Published:** 2025-10-10

**Authors:** Elise Sellars, Bonamy R. Oliver, Patty Leijten, Lucy Bowes

**Affiliations:** 1 Department of Experimental Psychology, Anna Watts Building, Radcliffe Observatory Quarter, University of Oxford , Oxford, OX2 6GG, UK; 2 IOE-Psychology & Human Development, UCL Institute of Education, University College London , London, UK; 3 Research Institute of Child Development and Education, University of Amsterdam , Amsterdam, The Netherlands

**Keywords:** ALSPAC, friendship, maltreatment, resilience, trajectories

## Abstract

Child maltreatment increases the risk of emotional and behavioral problems, yet many children demonstrate resilience, functioning better than expected given their level of maltreatment exposure. Although resilience is a dynamic process shaped by children’s social support, including friendships, how different patterns of resilience and friendship support unfold together across development remains unclear. To better understand this process, we examined how patterns of emotional resilience, behavioral resilience, and friendship support co-develop across childhood and adolescence. We used group-based multi-trajectory modeling with data from the Avon Longitudinal Study of Parents and Children (*N* = 6, 518, 51% female) to identify distinct patterns of emotional and behavioral resilience (doing better-than-expected given their level of maltreatment exposure) and friendship support, across five timepoints from ages 6 to 17 years. We identified five trajectory groups. Nearly half the sample maintained high emotional and behavioral resilience and friendship support across development. While resilience trajectories varied, friendship support was generally high across groups. Most children followed trajectories of high resilience and perceived friendship support. Even among children with lower emotional and/or behavioral resilience trajectories, friendship support remained high, an encouraging finding. Future research should examine how children’s other relationships (e.g., with parents and siblings) unfold alongside resilience.

## Introduction

Child maltreatment, including neglect and emotional, physical, or sexual abuse, is a well-established risk factor for significant and enduring mental health problems (Baldwin et al., 2023; Degli Esposti et al., 2020; Herbert et al., 2023). Indeed, up to a quarter of cases of some mental health conditions, such as anxiety, may be attributed to child maltreatment (Burghart & Backhaus, 2024). Most maltreatment is perpetrated by parents, with common behaviors involving physical and emotional abuse within the home (Devries et al., 2018). Despite the pervasive effects of maltreatment, outcomes vary considerably, with some children doing better-than-expected given their level of maltreatment exposure i.e., demonstrating resilience (Cicchetti, 2013; Collishaw et al., 2007).

Resilience is understood as a dynamic process shaped by an individual’s interactions with their environment, including factors such as friendship support (Fritz, de Graaff, et al., 2018; Kalisch et al., 2017). While existing research indicates that friendship may buffer against the detrimental effects of early life adversity on later mental health (van Harmelen et al., 2016, 2017), this likely represents only a partial understanding of a more intricate dynamic. For example, peer-influence theories suggest that friendships with peers with mental health problems may negatively influence one’s own mental health, due to issues such as negative friend dynamics or peer contagion (Dishion & Tipsord, 2011). Furthermore, resilience is a dynamic process, such that it is important to study the co-occurrence of relevant child characteristics (i.e., emotional and behavioral problems) and environmental factors (friendship support) over time, rather than simply assessing the predictive power of one factor on another. This is especially relevant as factors such as friendship support may fluctuate over time, either enhancing or diminishing resilience (Kalisch et al., 2017). Consistent with this, research has demonstrated that resilience and friendship support change together during adolescence. For example, van Harmelen et al. (2021) found that changes in friendship support and resilience (doing better-than-expected in terms of psychosocial functioning across several measures) from age 14 to 17 were positively correlated. However, little is known about how resilience and friendship support evolve together earlier in development, and over longer time periods, for example, across childhood and adolescence. Therefore, this study aimed to identify co-occurring developmental trajectories of children’s emotional and behavioral resilience (doing better-than-expected given their level of maltreatment exposure) and friendship support, using data from the Avon Longitudinal Study of Parents and Children (ALSPAC). Studying co-occurring trajectories across development also enables the potential identification of developmental cascades. This is a process through which early experiences (e.g., friendship support) create ripple effects across various domains (e.g., resilience to mental health problems), altering the course of development (Masten & Cicchetti, 2010).

### Resilience

Resilience research adopts a multisystemic perspective, defining resilience as “the process of multiple biological, psychological, social, and ecological systems interacting in ways that help individuals to regain, sustain, or improve their mental wellbeing when challenged by one or more risk factors” (Ungar & Theron, 2020, p. 1). Resilience is thus not a stable individual trait, but rather emerges from an individual’s experience with multiple, time-varying, and interacting systems (Kalisch et al., 2017). As such, low resilience at one timepoint does not necessarily preclude future resilience, and vise versa. Additionally, resilience is multidimensional, meaning an individual may exhibit resilience in one domain of functioning following adversity, but not another (Luthar & Cicchetti, 2000). Therefore, to enable a comprehensive understanding of an individual’s functioning, it is crucial to study resilience across multiple domains (e.g., in this study- emotional *and* behavioral problems).

Ways to conceptualize and measure resilience differ (Klika & Herrenkohl, 2013). Some research utilizes questionnaires that aim to directly quantify resilience as a measure of stress coping ability, for example, the Connor–Davidson Resilience Scale (Connor & Davidson, 2003) and the Brief Resilience Scale (Smith et al., 2008). While useful for assessing current functioning, such measures cannot fully capture a central part of contemporary conceptualizations of resilience, which understands resilience as a dynamic process of adaptation to adversity across an individual’s life- i.e., an individual’s functioning relative to their level of adversity (Kalish et al., 2017). Assessing resilience through this lens inherently requires measuring both an individual’s level of exposure to an earlier adversity and a measure of their current functioning. Therefore, to capture this dyadic element of resilience, we used a residuals approach, whereby “resilience” is conceptualized as doing better than expected given a level of exposure to adversity. In this study, better-than-expected refers to emotional or behavioral functioning given an individual’s level of exposure to maltreatment. Such an approach removes the need for arbitrary categorization of resilience versus vulnerability, looking instead at full range of functioning. This approach has been applied in research examining resilience to adversities such as peer victimization (Bowes et al., 2010), sibling victimization (Sellars et al., 2024), parental depression (Padaigaitė-Gulbinienė et al., 2024), and harsh parenting (van Harmelen et al., 2017). Recent research confirms the construct and predictive validity of this approach for assessing resilience to psychopathology in the ALSPAC cohort (Cahill et al., 2022).

### Role of friendship support

Resilience research has identified protective factors linked to better-than-expected outcomes following adversity, including individual-level (e.g., high self-esteem), family-level (e.g., high family cohesion), and community-level (e.g., high social support) factors (Fritz, de Graaff, et al., 2018). One identified factor is friendship support, which refers to an individual’s perception of the number of friends they have, and the degree to which individuals feel cared for, supported, and accepted by their friends (Reblin & Uchino, 2008).

Friendships play a crucial role in promoting children’s social and emotional development (Hartup, 2022). This may be particularly important for maltreated children, as several studies highlight that friendship helps mitigate vulnerability to psychopathology following childhood adversity. For example, van Harmelen et al. (2016) demonstrated the importance of positive social environments for adolescents who had experienced earlier adversities, including maltreatment – finding that friendship support at age 14 was associated with lower subsequent risk of depression at age 17 in adolescents exposed to early life stressors. Building on this work, van Harmelen et al. (2017) showed that friendship support during adolescence was associated with adolescent’s better-than-expected psychosocial functioning (i.e., resilience) following a range of early life negative family experiences, including parental abuse. Specifically, in a sample of 14–24 year olds, friendship support positively predicted resilient functioning three years later. In a further study, van Harmelen et al. (2021) found that improvements in friendships between ages 14 to 17 were associated with corresponding increases in resilience during the same period. Evidence from longitudinal birth cohort studies also demonstrates the importance of friendships. For example, in the ALSPAC cohort, higher levels of supportive peer relationships at age 15 were associated with lower levels of depression at age 18, including amongst those who experienced childhood emotional neglect (Glickman et al., 2021). Similarly, Cahill et al. (2023) found that high levels of friendship support at age 12 were associated with increased odds of belonging to a developmental trajectory characterized by resilience to adverse childhood experiences.

The mechanisms through which supportive friends enhance resilience are not yet fully understood. One possibility is that friendships provide opportunities to update self-cognitions (how an individual thinks about themselves, including for example, attributes they would use to describe themselves; van Harmelen et al., 2010), which may be harmed following child maltreatment (van Harmelen et al., 2016). Negative self-cognitions mediate the relationship between childhood maltreatment and poor mental health (van Harmelen et al., 2010). As such, friendships may foster more positive self-cognitions by increasing self-esteem and feelings of self-efficacy (Bolger et al., 1998; Fitzpatrick & Bussey, 2014). Additionally, friendships may help mitigate stress responses (Masten et al., 2012), and promote adaptive behaviors such as help-seeking and coping (Gunnar, 2017).

While the presence of friendships is associated with better-than-expected mental health following earlier adversities such as maltreatment, a lack of friendship may increase psychopathology risk, particularly among maltreated children, who face heightened challenges in forming friendships (8; Dodge et al., 1994; Rogosch & Cicchetti, 1994). Maltreated children may be especially vulnerable to “social thinning”, where their network of supportive relationships is either not fully established or diminishes over time (McCrory et al., 2022). This likely arises from several interconnected factors. For example, attachment theory posits that maltreatment may increase children’s likelihood of developing internal working models of relationships that are characterized by rejection and mistrust, thereby hindering the development of later friendships (Cyr et al., 2010). Difficulty forming friendships may also be compounded by fewer opportunities within maltreating home environments to learn prosocial interaction skills. The implicit interpersonal grammar hypothesis (Dishion, 2016) further explains that maltreatment experiences might shape children’s peer interactions directly through learned aggression and via a “grammar of coercion” (Dishion, 2016, p. 56). This involves the development of implicit beliefs that close relationships are untrustworthy and aggressive. Consequently, when children with these expectations interact with peers, they may be more likely to exhibit aggression, thereby impeding the development of supportive friendships. Thus, through a combination of these factors, a transactional cascade might be initiated, in which an absence of supportive friendships increases latent risk for psychopathology, further impairing children’s likelihood of forming friendships in a negative cycle (Viding et al., 2024).

### Research gaps in the study of resilience and friendship support

While resilience research has typically conceptualized friendship support as associated with better-than-expected outcomes, and its absence with worse outcomes, this relationship is likely to be complex. Peer-influence studies provide examples of the “dark side” of friendship support for children’s emotional and behavioral outcomes. For example, peer contagion is a mutual-influence process that occurs between an individual and a peer, which includes behaviors and emotions that may undermine an individual’s development or cause harm to others (Dishion & Tipsord, 2011). One key mechanism of peer contagion is “deviancy training,” where deviant behaviors (e.g., rule-breaking and aggression) are encouraged through positive reinforcement within friendships, such as mutual encouragement and normalization of such behavior (Dishion & Tipsord, 2011). There is a substantial body of literature in support of the concept of deviancy training. For example, friendships characterized by deviant stories, endorsements of deviant attitudes and norm-violating behavior predict growth in outcomes such as substance abuse, aggression, and intimate partner violence (Ha et al., 2023; Patterson et al., 2000; Piehler & Dishion, 2007).

Peer contagion also extends to emotional domains, such as depressive symptoms. Mechanisms driving this contagion include co-rumination (the excessive discussion of problems within the interpersonal context), which may amplify depressive tendencies within peer groups (Stevens & Prinstein, 2005). For example, higher levels of co-rumination within friendships are bidirectionally associated with higher levels of internalizing symptoms (Rose, 2021). Thus, peer-influence theories underscore the necessity of adopting a novel approach to the study of resilience and friendship support, one which acknowledges that friendship support is not always beneficial, to advance our understanding of resilience processes.

Furthermore, to advance the existing resilience literature, longitudinal studies are needed that specifically focus on friendship support and emotional and behavioral resilience following earlier maltreatment. While prior research has provided important insights into the role of friendship support following a range of adverse childhood experiences (e.g., parenting style, bereavement, maltreatment, and peer victimization; Cahill et al., 2023; van Harmelen et al., 2016, 2017), it has not focused exclusively on maltreatment. Studying functioning following exposure to a specific adversity (i.e., maltreatment) rather than several adversities grouped together can help to identify groups of children who might benefit from targeted interventions to support their wellbeing. Relatedly, prior studies used composite measures of psychosocial functioning in the generation of resilience scores (e.g., Van Harmelen et al., 2017, 2021). While composite scores increase parsimony and statistical power, it is also valuable to examine resilience in specific domains, such as emotional and behavioral problems. Analyzing these domains individually allows for the detection of meaningful differences in emotional and behavioral adaptation that might be otherwise obscured in a composite score, providing a more nuanced understanding of resilience. This is particularly relevant as peer contagion theories suggest that some friendships may be associated with increased difficulties in a specific mental health domain (Dishion and Tipsord, 2011), underscoring the importance of assessing resilience in ways that capture these distinctions.

Additionally, most studies focused on friendship support during adolescence (e.g., Cahill et al., 2023; Glickman et al., 2021; van Harmelen et al., 2016), given that it is a critical period of heightened peer influence (Collins & Laursen, 2000). However, friendships are also important for children’s earlier development (Dishion & Tipsord, 2011). van Harmelen et al. (2021) demonstrated the interplay between friendship support and resilience from ages 14–17, however is not known how friendship and resilience change together from childhood to adolescence. This is particularly important because friendship support can fluctuate across key transition periods, such as the move from primary to secondary school, where friendship stability is often low (Ng-Knight et al., 2019). To date, how such changes relate to resilience has not yet been explored.

Finally, previous studies have typically used variable-centered analyses, reporting population averages in mental health and friendship variables. Importantly, individual differences and distinct patterns over time are not captured with this approach. Yet, one of the hallmarks of outcomes following adversity is multifinality- the diversity of outcomes following an adverse event (Masten, 2024). Peer contagion literature also suggests that friendship support from peers with high levels of disruptive behavior/ depressive symptoms may increase vulnerability to mental-health difficulties for some children (Dishion et al., 1996; Rose, 2021). Person-centered approaches, such as group-based multi-trajectory modeling (Nagin et al., 2018), are particularly suited to identify patterns of development. For example, group-based multi-trajectory modeling may be a particularly suitable method for capturing the diversity of possible developmental patterns, as it identifies subgroups within a population that follow similar trajectories for key variables (e.g., emotional resilience, behavioral resilience, and friendship support) over time.

### The present study

Using group-based multi-trajectory modeling with ALSPAC data, we aimed to identify variation in co-occurring developmental trajectories of children’s emotional and behavioral resilience (doing better-than-expected given their level of maltreatment exposure) and perceived friendship support. Identifying these trajectories may offer valuable insights into how emotional and behavioral resilience and friendship co-develop across childhood and adolescence. Given the novelty of this approach, analyses were exploratory and data driven. Nonetheless, based on existing resilience research and peer-influence theories, we hypothesized that up to four distinct subgroups would emerge: (1) high emotional and behavioral resilience and high friendship support; (2) low emotional and behavioral resilience and low friendship support; (3) low behavioral resilience, but high emotional resilience and high friendship support (following peer deviancy literature); and (4) low emotional resilience, but high behavioral resilience and high friendship support (following emotional contagion theory).

## Methods

### Data source

ALSPAC is an ongoing population-based birth cohort study, designed to investigate influences on health and development across the life course (Boyd et al., 2013; Fraser et al., 2013). Pregnant women resident in Avon, UK, with expected dates of delivery between 1st April 1991 and 31st December 1992 were invited to take part in the study. The initial number of pregnancies enrolled was 14,541, with 13,988 children alive at 1 year of age. Please note that the study website contains details of all the data that is available through a fully searchable data dictionary and variable search tool (http://www.bristol.ac.uk/alspac/researchers/our-data/). Ethical approval for the study was granted by the ALSPAC Ethics and Law committee and the Local Research Ethics Committees. Informed consent for the use of data collected via questionnaires and clinics was obtained from participants following the recommendations of the ALSPAC Ethics and Law Committee at the time.

### Participants

The analytic sample included 6,518 children who met our inclusion criteria: (1) data were available on child maltreatment for at least one of six possible timepoints between 8 months and 6 years (to maximize sample size); and (2) data on emotional problems, behavioral problems, and friendship support (trajectory variables) for at least two of the possible five timepoints (from approximately 6 to 17 years old) for each measure, consistent with previous studies utilizing trajectory modeling (Holst et al., 2023; Nivard et al., 2017). Compared to children in the analytic sample, excluded children (*N* = 7, 429) were more likely to be male, of non-White ethnicity, and have parents who were younger, with a lower socio-economic status, and higher levels of mental health difficulties (Table S1, supplemental material).

### Measures

#### Emotional and behavioral resilience

We created measures of emotional and behavioral resilience using a residuals approach, which captures the extent to which an individual has better-than-expected, or worse-than-expected functioning, given their level of exposure to maltreatment. This section outlines the measures which composed our emotional and behavioral resilience variables (a. Maltreatment; b. Emotional and behavioral problems); and then explains how we used these measures to create the residuals scores which formed the emotional and behavioral resilience variables entering the trajectory analyses (c. Emotional and behavioral resilience – a residuals approach).

##### Maltreatment

Exposure to maltreatment was assessed through maternal reports of physical or emotional cruelty towards the child (yes/no), perpetrated by either the mother or her partner. Data were collected via postal questionnaires at six timepoints when children were approximately 8 months, 1 year 9 months, 2 years 9 months, 3 years 11 months, 5 years 1 month, and 6 years 1 month old. The first six years of life were chosen as the exposure period because this is a critical time when the risk of maltreatment is highest, and early childhood maltreatment is known to increase vulnerability to later psychopathology (Dunn et al., 2020; Jaffee, 2017).

In line with previous studies (e.g., Dunn et al., 2024; Khambati et al., 2018), at each timepoint and for each maltreatment category (emotional or physical), we considered children maltreated if the mother responded “yes” to either her or her partner perpetrating that category of maltreatment. Therefore, exposure to either physical or emotional maltreatment was defined as at least one affirmative response by the mother in that particular maltreatment category, regardless of the perpetrator (s) identified. To operationalize this, two binary variables were created for each timepoint: one for physical maltreatment and one for emotional maltreatment, where 0 = *no maltreatment* and 1 = *maltreated*. To ensure conservative coding, if a child had data on one maltreatment category but was missing data for the other maltreatment category at a given timepoint, the missing maltreatment category was coded as 0.

We then created a cumulative maltreatment score by summing the two binary variables (emotional and physical maltreatment) across all six timepoints. The maximum possible score was 12 (indicating reports of both emotional and physical maltreatment at all timepoints) and a minimum score of 0 (indicating no report of maltreatment at any timepoint). Using a cumulative score maximized the variance available for generating the resilience to emotional and behavioral problems variables used in the main analyses.

##### Emotional and behavioral problems

Emotional problems and behavioral problems were assessed using maternal reports from the Strengths and Difficulties Questionnaire (SDQ; Goodman, 1997), a widely used instrument with established reliability and validity, including in community samples. Data were collected via postal questionnaire at five timepoints when the children were approximately 6 years and 8 months, 9 years, 11 years, 13 years, and 16 years old. These timepoints were chosen as they follow the maltreatment exposure period, and represent the maximum number of timepoints across middle childhood to adolescence which approximately match the available assessment timepoints for friendship support.

Mothers rated their child’s behavior over the past six months on a three-point scale (0 = *not true*, 1 = *somewhat true*, or 2 = *certainly true)*. Following established guidelines (https://www.SDqinfo.org/py/SDqinfo/c0.py) behavioral problems (i.e., externalizing problems) scores were calculated by summing the conduct problem subscale (five items, e.g., “Often has temper tantrums”) and hyperactivity and inattention subscale (five items, e.g., “Restless, overactive, cannot stay still for long”), with a maximum score of 20. At each timepoint, if an individual was missing one of the subscales, their behavioral problems score was coded as missing.

Emotional problems scores were measured using the emotional problems subscale, which captures affective symptoms (five items, e.g., “Often seems worried”), with a maximum score of 10. To avoid confounding with the friendship support measure, the emotional problems subscale was not combined with the peer problems subscale (commonly combined to form an internalizing problems subscale). For both emotional and behavioral problems, higher scores indicated greater difficulties.

##### Emotional and behavioral resilience – a residuals approach

We used a residuals approach to assess an individual’s emotional and behavioral resilience given their level of maltreatment exposure. Conceptually, this approach decomposes variance in outcome variables (in this study, emotional and behavioral problems scores) into two components: (1) the variance explained by exposure to a particular adversity (in this case, child maltreatment) and (2) the residual variance, which is independent of exposure to the measured adversity. The residual component captures individual differences in the outcome variable which are not explained by exposure to the measured adversity. As such, the residuals score reflects a full range of functioning, indicating the extent to which a cohort member has worse, or better, outcomes than predicted given their level of exposure to child maltreatment. This forms our measure of emotional and behavioral resilience. Please see Bowes et al. (2010), Cahill et al. (2022) and Ioannidis et al. (2020) for comprehensive overviews of this method.

A key strength of the residuals approach is that it accounts for variation in adversity exposure, aligning with the principle that resilience must be assessed relative to adversity (Masten, 2024). For example, two children may both show moderate levels of emotional difficulties, but if only one child has experienced severe maltreatment, then a residuals approach would identify this child as demonstrating resilience (i.e., better-than-expected functioning given their maltreatment exposure), a distinction missed using raw scores alone (Ioannidis et al., 2020). To capture this nuance, our analytic sample included children across the full spectrum of maltreatment exposure, from none to severe, rather than restricting analyses to a subset of maltreated children. This not only allowed for the identification of children functioning better- or worse-than-expected for their level of adversity but also ensured sufficient variation in residuals scores to meaningfully measure emotional and behavioral resilience.

We generated residuals scores for both emotional and behavioral problems at each of the five timepoints. To illustrate, outlined here is an example of how resilience to emotional problems was calculated per timepoint: First, emotional problem scores for that timepoint were regressed on the cumulative maltreatment exposure scores (using a linear model) and the residuals were extracted. Residuals were then reverse coded, so that positive residuals indicated cohort members with fewer emotional problems than expected given their exposure to child maltreatment – i.e., demonstrating higher emotional resilience at this timepoint. Conversely, negative residual scores indicated children with greater than expected levels of emotional problems, reflecting vulnerability- i.e., lower level of emotional resilience. This process was conducted for each of the five emotional problems timepoints, and resulting residual scores were used in trajectory analyses as indicators of emotional resilience at each timepoint. The same procedure was applied to behavioral problems scores to generate behavioral resilience trajectory variables. Linear models were used across all timepoints.

#### Friendship

Children reported perceived friendship support using the shortened (five-item) version of the Cambridge Hormones and Moods Project Friendship questionnaire (Goodyer et al., 1990). Data were collected during clinic visits at five timepoints when the children were approximately 8 years, 10 years, 12 years and 6 months, 13 years and 6 months, and 17 years and 6 months old.

Children were asked to rate the availability and quality of their friendships, using a four-point Likert scale: “Are you happy with the number of friends you’ve got” (0 = *unhappy*, 1 = *quite unhappy*, 2 = *quite happy*, 3 = *very happy*), “Do your friends understand you” (0 = *not at all*, 1 = *not often*, 2 = *sometimes*, 3 = *most of the time*); “Do you talk to your friends about problems” (0 = *not at all*, 1 = *not often*, 2 = *sometimes*, 3 = *most of the time*); “Do you see your friends outside of school” (0 = *hardly ever*, 1 = *less than once per week*, 2 = *at least once per week*, 3 = *almost every day*) and “Overall how happy are you with your friends” (0 = *unhappy*,1 = *quite unhappy*, 2 = *quite happy*, 3 = *very happy*). In line with a previous ALSPAC study utilizing this measure (Glickman et al., 2021), the five items were summed to create a total score ranging from 0 to 15, with higher scores indicating better perceived overall quality of friendship. For individuals missing either one or two items at a particular timepoint (i.e., up to 40% item-level missingness; Perley-Robertson et al., 2024), an individual’s missing items were prorated as the mean of their available items before generating their total score. If an individual was missing more than two items at a particular timepoint, their total score for that timepoint was coded as missing.

#### Trajectory group characteristics

The following variables were used to describe trajectory groups, all collected through maternal self-report during pregnancy, except for child sex (from the birth certificate) and child birthweight (from medical records).

##### Family factors

**Binary indicators:**

**Socio-economic status:** a. Mother’s and partner’s highest educational qualifications, dichotomized into (i) O-Levels or higher (advanced-level qualifications, university degree, or ordinary-level qualifications) or (ii) lower than O-Levels (certificate of secondary school education, vocational, or none); b. Mother’s household social class, dichotomized into (i) high (professional, managerial, or skilled professions) or (ii) low (partly or unskilled occupations); c. Mother’s homeowner status, dichotomized into (i) mortgaged/owned or (ii) other (including rented).

**Maternal smoking:** Whether the mother had smoked tobacco during the first three months of pregnancy (yes/no).

**Maternal alcohol use:** Alcohol consumption during first three months of pregnancy (yes/no).

**Continuous indicators:**

**Depression (maternal and partner):** Assessed using the Edinburgh Postnatal Depression Scale (Cox et al., 1987) with scores ranging from 0 to 30 (higher scores indicate more depressive symptoms).

**Maternal anxiety:** Assessed using the anxiety items from the Crown-Crisp Experiential Index (Birtchnell et al., 1988), with scores ranging from 0 to 16 (higher scores indicate more anxiety symptoms).

**Maternal age:** Mother’s age at delivery.

##### Child factors

**Binary indicators:**

**Sex:** Female or male.

**Ethnicity:** White or non-White.

**Continuous indicator:**

**Birthweight:** Measured in grams.

#### Analytic strategy

Our research questions, hypotheses, and analysis plan were pre-registered on the Open Science Framework (https://osf.io/9kp2b). Group-based multi-trajectory modeling was used to identify distinct trajectories of emotional and behavioral resilience (given level of exposure to maltreatment) and friendship support. This person-centered approach, an application of finite mixture modeling, identifies clusters (i.e., groups) of individuals with similar trajectories across multiple repeated measures. Trajectory groups are not literal entities but represent key patterns within the study population. The goal is to identify the smallest number of groups that capture distinctive features of the study population-conceptualized as longitudinal latent strata (Nagin et al., 2018, 2024).

Trajectory models included three indicator variables, each assessed at five timepoints (henceforth referred to as T1–T5) from childhood to adolescence: (1) emotional resilience (i.e., emotional problems residuals scores- as outlined in the “measures” section); (2) behavioral resilience (i.e., behavioral problems residuals scores); and (3) perceived friendship support. This allowed trajectories of resilience and friendship to be jointly modeled across development. Continuous scores were used for all indicator variables.

To determine the optimal number of trajectory groups, models were run with increasing numbers of groups (1–9; a ten-group model did not converge). For each model, cubic, quadratic, and linear functions were evaluated. For all models, quadratic functions fit the data best. Model selection was based on established model fit indices, such as the sample size adjusted Bayesian information criterion, with smaller values (i.e., values closest to 0) indicating a better fit (Nagin, 2005). Model adequacy was assessed using average posterior probabilities and odds of correct classification. Following established guidelines (Klijn et al., 2017; Nagin et al., 2018), models with average posterior probability values greater than 0.70 and odds of correct classification values greater than 5.0 were considered good fits. Model interpretability and subgroup size was also considered. Analyses were conducted using the “traj” procedure in Stata (Jones & Nagin, 2013). No variables were controlled during trajectory derivation, as doing so could affect true subgroup formation and classification. Missing data were handled using full information maximum likelihood. As this method for handling missing data assumes that data is missing at random, prior to running trajectory analyses, we conduct logistic regressions to determine whether missingness in each of the study’s indicator variables was associated with baseline sample characteristics- i.e., missingness was explained by observed variables. Analyses confirmed that missingness for each indicator variable significantly correlated with multiple baseline variables, supporting the plausibility of the missing at random assumption, and the appropriateness of using full information maximum likelihood to address missing data.

The following results section reports the probability of trajectory group membership, indicating the proportion of the population in each trajectory group, and describes group characteristics (percentages and means).

## Results

### Descriptives

The analytic sample included 6,518 children. Table 1 shows their socio-demographic and baseline parental factors.

Consistent with other ALSPAC studies measuring maltreatment in early childhood (Dunn et al., 2024; Khambati et al., 2018), 12.1 % of the sample (*n* = 786) experienced any type of maltreatment at at least one timepoint. Among those who experienced maltreatment, 1.2% experienced physical maltreatment only, 8% experienced emotional maltreatment only, and 2.9% co-occurring physical and emotional maltreatment.

The cumulative maltreatment scores ranged from 0 to 10 (*M* = 0. 25, *SD* = 0. 86). Please see Table S2, supplemental material, for the full distribution of these scores (*n* and percentages). For the 12.1% who scored > 0 (i.e., experienced maltreatment at at least one timepoint), scores of 1 (6.5% of the sample) and 2 (2.5% of the sample) were the most common.

For each timepoint at which emotional and behavioral problems were assessed (T1–T5), maltreatment exposure was associated with higher levels of emotional and behavioral problems (*p* <.001; supplemental material, Table S3). Descriptive statistics for the indicator variables entering the trajectory models (i.e., emotional resilience, behavioral resilience, and friendship support) are also available in the supplemental material (Table S4).

### Main analyses

To identify the best fitting model for number of trajectory groups, models were run with one to nine classes. Fit statistic values (Table 2) indicated small, incremental improvements in model fit with increases in number of trajectory groups, with all models far exceeding minimum values for model adequacy statistics. Therefore, selection of a parsimonious model was prioritized (Nagin, 2005). A five-group model was selected as the most parsimonious model which captured meaningfully distinct trajectory groups, while avoiding subgroups with very small proportions (e.g., a six-trajectory group model contained a subgroup with just 2% of the sample).

Model adequacy statistics suggest that a five-group model fit the data well, with each group’s average posterior probability and odds of correct classification value far exceeding 0.70 and 5, respectively (Table 3).

**Figure 1:**
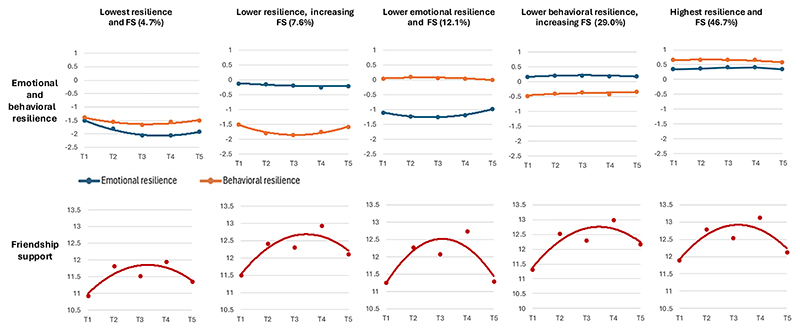
Trajectories for the best fitting (five-group) model. *Note*. FS = friendship support. Positive residuals scores indicate higher levels of resilience, negative scores indicate lower resilience. Higher friendship support values indicate higher overall quality of friendship.

Figure 1 shows the five groups of co-occurring trajectories of friendship support and emotional and behavioral resilience given level of exposure to maltreatment. The group labels “higher” or “lower” qualitatively describe relative differences in trajectories, rather than absolute cut-offs or categories of functioning. For example, while positive residuals scores indicate greater resilience, and negative scores lower resilience, there are no predefined thresholds for “high” or “low” resilience. Similarly, higher friendship support scores indicate better perceived friendship quality, though no established cut-offs define “high” or “low” support. Notably, average friendship scores across all timepoints and subgroups range from 10 to 14 (out of 15). Therefore, groups labeled as having “lower” friendship support are only lower in comparison to other trajectory groups.

The largest group (46.7%, *n* = 3, 041) showed high emotional and behavioral resilience alongside high levels of friendship support (“Highest resilience and FS”). This group consistently demonstrated the highest levels of all indicator variables across all timepoints. The second largest group (29.0%, *n* = 1, 888) showed low behavioral resilience but moderate emotional resilience, with increasing friendship support (“Lower behavioral resilience, increasing FS”). Another mixed-resilience group (12.1%, *n* = 786) showed the opposite resilience pattern- with low emotional resilience, moderate behavioral resilience, and lower friendship support (“Lower emotional resilience and FS”). A smaller group (7.6%, *n* = 497) showed low emotional resilience, very low behavioral resilience, and increasing friendship support (“Lower resilience, increasing FS”). The smallest group (4.7%, *n* = 306) showed very low emotional and behavioral resilience and the lowest levels of friendship support (“Lowest resilience and FS”).

Descriptive statistics (*M, SD* and %s) for socio-demographic and baseline parental factors across different trajectory groups are presented in Table 4. The most pronounced difference was child gender: “Lower resilience, increasing FS” group, with particularly low behavioral resilience, had the highest proportion of males (67%), while the “Lower emotional resilience and FS” group had the lowest proportion of males (32%). In addition, there were some small differences in family characteristics between groups. For example, the “Lowest resilience and FS,” and “Lower resilience, increasing FS” groups had families with indicators of lower socio-economic status and higher levels of parental mental health problems, maternal smoking and alcohol consumption, with these factors the most pronounced in the “Lowest resilience and FS” group. In contrast, the “Highest resilience and FS” group had families with the highest average socio-economic status (e.g., 15% of mothers with a low education level, compared to 29% in the “Lowest resilience and FS” group) and the lowest parental mental health problems.

## Discussion

This study examined co-occurring trajectories of emotional and behavioral resilience (considered as better-than-expected functioning given level of maltreatment exposure) and friendship support, from ages 6 to 17 years. Five distinct subgroups were identified. Resilience patterns varied across groups, consistent with the concept of multifinality (Masten, 2024) and the domain-specific nature of resilience (Luthar & Cicchetti, 2000). In contrast, there was little variation in friendship support trajectory patterns: overall, levels of perceived support were high across groups, with minimal variation between groups (i.e., some groups with a trajectory of slightly lower support, or small increases in perceived support over time).

Most children showed high emotional and behavioral resilience and friendship support. That almost half of the children in the sample showed this pattern of resilience aligns with heartening findings that resilience across domains is the most common response to adversity (Bonanno, 2021; Collishaw et al., 2007; Masten, 2014), while emphasizing the need to better understand when this is not the case.

Two groups of children showed mixed patterns of resilience. Children in one group exhibited low emotional resilience but relative behavioral resilience and lower friendship support (“Lower emotional resilience and FS,” 12.1%). Children in the other subgroup showed low behavioral resilience but higher emotional resilience and increasing friendship support (“Lower behavioral resilience, increasing FS,” 29.0%). Peer-influence theories may help explain these patterns. For example, the persistent low behavioral resilience of children in the “Lower behavioral resilience, increasing FS” group, despite small rises in friendship support, might reflect deviancy training, particularly as 57% of this group were boys, and this process is well-documented in male friendships (Dishion et al., 1996; Poulin et al., 1999). Conversely, the low emotional resilience of children in the “Lower emotional resilience and FS” group, despite average friendship scores at each timepoint exceeding 10 (out of a possible 15), may stem from co-rumination (Rose, 2002). This process is more common in girls, and nearly three-quarters of this group were female (Table 4). However, like many cohort studies, ALSPAC does not include data on friends’ emotional or behavioral functioning, so these interpretations are only speculative. Future research should incorporate peer-level data into trajectory models to help clarify how friends’ behavior shapes resilience processes.

Some children showed an unexpected pattern of very low behavioral and low emotional resilience, with increasing friendship support (“Lower resilience, increasing FS” group (7.6%)). One potential explanation for this is that these children’s support needs may be greater than can be met by the benefits of increasingly supportive friendships. This aligns with a multisystemic view of resilience, which emphasizes the necessity of support systems at multiple levels of the socio-ecological system (e.g., family, school, wider community) to facilitate children’s wellbeing (Masten et al., 2021; Ungar & Theron, 2020). Given the increased vulnerability of boys to deviancy training (Dishion et al., 1996; Poulin et al., 1999), the high proportion (67%) of boys in this group could suggest that deviancy training is a contributing factor, particularly to their low behavioral resilience. Nevertheless, it is likely that multiple factors are involved. Whilst this pattern could equally reflect gender differences in prevalence of mental health problems unrelated to social support, where girls show higher rates of internalizing problems, and boys higher externalizing problems (Knowles et al., 2025; Yang et al., 2024), additional sensitivity analyses suggest this is unlikely to be the case (see Supplemental material, Table S5).

Most baseline characteristics were similar across groups (Table 4). However, children in the two trajectory groups with the lowest levels of resilience had the highest levels of potential stressors, such as lower socio-economic status and elevated parental mental health problems. While these findings are descriptive and should not be overinterpreted, they align with evidence that adverse childhood experiences often co-occur (Brown et al., 2019). In the context of such cumulative stressors, individual protective factors (e.g., friendship support) may be insufficient to facilitate resilience (Jaffee et al., 2007; Vanderbilt-Adriance & Shaw, 2008).

In terms of developmental trends, friendship support declined across all groups between T2 (10 years) and T3 (12 years), and again from T4 (13 years 6 months) to T5 (17 years). These decreases align with educational transitions in England, where children move from primary to secondary school, and then to further education. Such transitions are periods of heightened friendship instability (McMillan et al., 2025). For example, fewer than a quarter of children maintain the same best friend across the primary- to-secondary school transition (Ng-Knight et al., 2019). While ALSPAC does not directly assess friendship stability, stable friendships are associated with better friendship quality (Poulin & Chan, 2010). This suggests that the small declines in friendship support evident in the trajectory groups may reflect normative shifts in friendship stability.

Despite minor fluctuations (e.g., some groups with lower/increasing trajectories), friendship support was generally high across subgroups, with no evidence of the negative developmental cascades that maltreated children may be at risk of (Viding et al., 2024). There are several possible explanations for this. One possibility is that children genuinely perceived high levels of friendship support across development, even among those with lower levels of resilience. In the context of maltreatment, this is particularly encouraging, suggesting that not all maltreated children perceive friendship difficulties. Alternatively, it may be that the measure did not sufficiently capture variation in children’s perceptions of friendships. For example, other aspects of friendship (e.g., reciprocity), not assessed in ALSPAC, might show greater variation across subgroups. For example, reciprocal friendships may be especially important for maltreated children, offering a sense of security and self-worth that maltreatment undermines (van Harmelen et al., 2010). Reciprocated friendships may also provide opportunities to learn and practice social skills not taught at home (Lansford et al., 2003). Nevertheless, meta-analytic evidence indicates that perceived friendships may still be a strong predictor of mental health outcomes, even in the absence of reciprocity data (Schwartz-Mette et al., 2020).

Additionally, beyond perceived friendship quality, friends’ behavior may have a stronger influence on an individual’s behavioral/emotional resilience, aligning with the peer-influence processes of deviancy training (Dishion & Tipsord, 2011) and co-rumination (Rose, 2021). Indeed, children whose friends exhibit internalizing/externalizing behaviors are more likely to report increases in these same behaviors over time (Giletta et al., 2021). Our friendship measure may not have fully captured these negative friendship dynamics, which might show greater variation between subgroups.

Using a residuals approach to measure resilience is a key strength of this study, with just one previous study using a residuals approach to plot resilience trajectories following early life adversity (Cahill et al., 2023). Because the residuals approach accounts for exposure to maltreatment, this allows for individuals with moderate outcomes following maltreatment to be included as demonstrating resilience, offering a more comprehensive understanding of emotional/behavioral functioning than traditional resilience definitions based on current functioning only (e.g., an absence of psychopathology; Klika & Herrenkohl, 2013).

A further strength of the residuals approach we used is its analysis of functioning within the full ALSPAC sample, encompassing a complete range of maltreatment exposure, including those unexposed. However, a potential limitation is that high resilience subgroups might simply contain higher numbers of non-maltreated children. Additional post-hoc (not pre-registered) analyses investigated this. Indeed, the “Highest resilience and FS” group had a lower percentage of maltreated children (11%), compared to other lower resilience groups (e.g., 21% in the “Lowest resilience and FS” group) (Table S6a, supplemental material). Encouragingly, however, focusing only on children exposed to maltreatment (*N* = 786; Table S6b supplemental material) within each subgroup shows that most maltreated children fell within the highest resilience subgroups, reinforcing the finding that resilience is the most common response following adversity. Moreover, these children had the highest cumulative maltreatment scores, indicating that the highest resilience subgroup does indeed contain children functioning better-than-expected despite maltreatment. Similar baseline child and family characteristics (per subgroup) across the whole sample (Table 4) and subsample of children exposed to maltreatment (Table S6b, supplemental material) further support studying resilience in the full sample. With key implications for future research, this suggests that children exposed to maltreatment are not a group with such distinct characteristics that they must be studied in isolation, rather they share similar baseline characteristics with the broader population.

More broadly, using person-centered methodology enabled the identification of more detailed patterns of resilience and friendship support, such as the two mixed resilience groups, that would likely be overlooked in a variable-centered approaches. This highlights nuances in how resilience and friendship support co-occur- for example, high friendship support can co-occur with low levels of resilience in one mental health domain, but relative resilience in another, a new finding for the literature. ALSPAC’s longitudinal data also allowed for tracking of resilience and friendship support across development, including key educational transitions, extending prior research that focused mainly on adolescence. Finally, use of multiple informants (self-reported friendship support, caregiver report of emotional and behavioral problems) reduced the risk of common-rater bias.

Study findings also have implications for future research. While friendships are important for children’s development, resilience arises from multiple, interacting systems (Fritz, Fried, et al., 2018; Ioannidis et al., 2020). For example, warm parent–child and sibling relationships also contribute to children’s resilience (Bowes et al., 2010). Although friendship support was high across all subgroups, this may not be the case for family relationships. While examining these relationships was beyond the scope of the present study, future research should consider how resilience trajectories co-occur with trajectories of parent–child and sibling relationships. However, few longitudinal datasets include repeated measures of adversity exposure, mental health, and family/peer relationship variables, limiting current ability to study these processes across development (Shanahan et al., 2024).

Additionally, the data used in this study were collected during the 1990s and 2000s. However, the prevalence and severity of emotional and behavioral difficulties in children have increased in recent decades (Armitage et al., 2024; Newlove-Delgado et al., 2022), as have levels of adolescence loneliness (Twenge et al., 2021). Furthermore, markers of friendship quality (e.g., how often cohort members meet up with their friends outside of school) may have changed since the ALSPAC data were collected, given that adolescents increasingly connect with friends via social media (Pouwels et al., 2021). Therefore, it will be important for future studies to assess whether this study’s findings replicate in a contemporary context, and to include measures of friendship support that capture aspects of interactions via digital devices.

Despite its strengths, this study has several limitations. First, generalizability of findings is limited by most participants in the ALSPAC cohort being of White ethnicity, and also of higher socio-economic status than the British population (Fraser et al., 2013). Additionally, as with all longitudinal studies, attrition may limit generalizability of the findings. Although full-information maximum likelihood was used to handle missing data in the included sample, it cannot account for excluded participants. Excluded participants had higher levels of baseline risk factors for later mental health problems (e.g., parental mental health problems; Rajyaguru et al., 2021); Table S1), so the identified resilience trajectories may not fully reflect the experiences of children growing up with more risk factors for mental health problems.

Second, because the residuals approach operationalizes resilience as better functioning than would be expected given an individual’s level of maltreatment, resulting residual scores are inherently tied to the prevalence of maltreatment within this sample. Therefore, resilience trajectories may not generalize to populations with different prevalence rates. However, as ALSPAC’s maltreatment prevalence rates are similar to other United Kingdom cohorts, such as the Millennium Cohort Study and the E-Risk Study (Farooq et al., 2024), findings may be generalizable at least within this context.

The measures used to generate resilience trajectory variables also have limitations, particularly regarding the assessment of maltreatment exposure. We used prospective maternal reports (six timepoints from 8 months – 6 years). This may have underestimated prevalence rates compared to retrospective self-report, due to parents underreporting due to fear of being reported to authorities (Baldwin et al., 2019). While retrospective self-report may capture cases not reported by parents, they are vulnerable to subjective interpretations of past experiences, and potential confounding by current mental health status (Baldwin et al., 2019, 2024). Although there is no single optimal method for assessing maltreatment, we prioritized prospective reports because repeated assessments enabled generation of a cumulative maltreatment score, ensuring more available variation when generating residuals. Alternative indicators of maltreatment in ALSPAC, such as child protection registration data, were deemed unsuitable, as they only capture the most severe cases (Sidebotham & Heron, 2006), and have high attrition (Khambati et al., 2018). ALSPAC’s retrospective measure (which asked cohort members at age 22 to recall maltreatment by family members before age 11) was not as appropriate for this study, because, as a single timepoint measure, it precluded generation of a cumulative score, as well as overlapping with the time period covered by trajectory variables.

Second, ALSPAC’s measures of maltreatment consisted of two items per timepoint, assessing whether parents were physically or emotionally cruel to their child. This might not capture the full range of specific maltreating behaviors (e.g., neglect, sexual abuse, or specific forms of psychological aggression; Backhaus et al., 2023). Therefore, it will be important for future studies examining trajectories of resilience given early life maltreatment to use more comprehensive instruments to assess maltreatment exposure.

Additionally, this study focused on maltreatment prior to age six, as this is a developmentally sensitive period during which maltreatment may be particularly harmful (Jaffee, 2017). However, maltreatment will continue throughout childhood for some children (Bigler et al., 2025). Therefore, unmeasured maltreatment, along with other adversities, may confound resilience measures. Finally, both maltreatment and child mental health were assessed using parent reports. Shared method variance could potentially inflate the associations between these variables when generating residual scores. Ideally, a mental health measure from a different source would have been used to mitigate this bias, however, such measures were not available at the timepoints necessary for our trajectory analyses.

To conclude, this longitudinal analysis extends the resilience and friendship support literature by examining co-occurring trajectories of emotional and behavioral resilience and friendship support from ages 6 to 17 years, contributing new insights into how these factors co-occur across development. While resilience patterns differed across groups, friendship support trajectories showed less variation, with relatively high levels across all groups. Most children followed trajectories of high resilience and friendship support, and even among those showing more vulnerable trajectories, perceived friendship support remained high- an encouraging finding. However, it remains to be determined whether this is also the case for other salient relationships in children’s lives, such as with parents or siblings.

## Figures and Tables

**Table 1:** Socio-demographic and baseline parental factors in the analytic sample (*N* = 6, 518)

Variable	% or *M (SD)*	Range (maximum)
**Child factors**		
Gender (male)	49.19	
Ethnicity (White)	96.39	
**Family factors**		
Maternal age at delivery (years)	29.22 (4.49)	<16 – >43
Maternal education (< O-levels)	17.99	
Paternal education (< O-levels)	22.36	
Household social class (low)	15.18	
Maternal homeownership status (mortgaged/owned)	84.23	
Maternal depression	6.34 (4.58)	0 − 28 (30)
Paternal depression	4.01 (3.77)	0 − 26 (30)
Maternal anxiety	4.67 (3.41)	0 − 16 (16)

*Note. M = mean, SD* = standard deviation. Range included the sample range and possible maximum score in parenthesis for continuous variables. Maternal and paternal education- O - levels (“Ordinary level” exams obtained by UK students at age 16). Maternal and paternal depression assessed using the Edinburgh Postnatal Depression Scale. Maternal anxiety assessed using the anxiety items from the Crown-Crisp Experiential Index.

**Table 2:** Model fit statistics

Number of groups	BIC_n	AIC	LL
1	–134443.73	–134403.04	–134391.04
2	–127991.80	–127917.19	–127895.19
3	–126068.06	–125959.54	–125959.54
4	–124024.55	–123882.12	–123840.12
5	–**123196.40**	–**123020.06**	–**122968.06**
6	–122629.30	–122419.05	–122357.05
7	–122271.25	–122027.09	–121955.09
8	–121910.41	–121632.34	–121632.34
9	–121582.70	–121270.71	–121178.71

*Note.* BIC_n Bayesian information criterion adjusted for number of observations; AIC Akaike information criterion; LL log-likelihood. Values for the selected five-group model are in bold. Fit indices are based on quadratic functions for all models.

**Table 3:** Model adequacy statistics (five-group model)

Trajectory group	*N* (%)	AvePP	OCC
#1	306 (4.7)	0.92	223.7
#2	497 (7.6)	0.90	107.7
#3	786 (12.1)	0.88	53.1
#4	1,888 (29)	0.86	15.6
#5	3,041 (46.7)	0.92	13.4

*Note.* AvePP average posterior probability; OCC odds of correct classification. Membership probability (AvePP) greater than 0.70 and OCC greater than 5 represent a good model fit.

**Table 4:** Descriptive statistics per trajectory group

Variable	Lowest resilience and FS (4.7%)	Lower resilience, increasing FS (7.6%)	Lower emotional resilience and FS (12.1%)	Lower behavioral resilience, increasing FS (29.0%)	Highest resilience and FS (46.7%)
**Child factors**					
Gender (% male)	50.33	67.20	32.19	57.63	45.28
Ethnicity (% White)	94.08	95.88	96.96	96.48	96.55
Birthweight in grams *(M, SD)*	3371.41 (529.64)	3390.69 (539.48)	3403.11 (570.30)	3408.57 (550.42)	3442.48 (529.34)
**Family factors**					
Maternal age at delivery (years) (*M, SD*)	28.14 (4.82)	28.41 (4.57)	29.20 (4.51)	28.96 (4.55)	29.63 (4.37)
Maternal education (% < O – levels)	28.67	24.63	17.43	19.39	15.18
Paternal education (% < O – levels)	29.25	29.52	22.44	24.60	19.30
Household social class (% low)	21.60	18.47	15.87	15.26	13.82
Maternal homeownership status (% mortgaged/ owned)	73.29	77.85	83.53	82.56	87.57
Maternal depression (*M, SD*)	9.10 (5.12)	7.78 (4.90)	7.44 (4.66)	6.62 (4.58)	5.58 (4.23)
Paternal depression (*M, SD*)	5.64 (4.39)	4.34 (3.92)	4.36 (3.73)	4.08 (3.66)	3.69 (3.70)
Maternal anxiety (*M, SD*)	6.57 (3.74)	5.58 (3.67)	5.58 (3.41)	4.82 (3.43)	3.99 (3.13)
Maternal smoking (% yes)	29.70	25.46	15.87	20.05	13.12
Maternal alcohol consumption (% yes)	52.17	57.46	57.44	59.36	54.92

*Note. M = mean, SD* = standard deviation, FS = Friendship support. Maternal and paternal education- O - levels (“Ordinary level” exams obtained by UK students at age 16). Maternal and paternal depression assessed using the Edinburgh Postnatal Depression Scale. Maternal anxiety assessed using the anxiety items from the Crown-Crisp Experiential Index.

## Data Availability

Access to ALSPAC data is through a system of managed open access (https://www.bristol.ac.uk/alspac/researchers/access/).

## Footnotes

**Supplementary material.** The supplementary material for this article can be found at https://doi.org/10.1017/S0954579425100758.
